# Toward a Typology of Patients With Head and Neck Cancer: Biopsychosocial Profiles Predicting Longitudinal Quality of Life

**DOI:** 10.1002/hed.70231

**Published:** 2026-03-11

**Authors:** Haley Deamond, Cyril Devault‐Tousignant, Jacob Lang, Christopher Lo, Jennifer Silver, Nader Sadeghi, Zeev Rosberger, Saul Frenkiel, Michael Hier, Anthony Zeitouni, Karen Kost, Alex Mlynarek, Keith Richardson, Gabrielle Chartier, Marco Mascarella, Khalil Sultanem, Georges Shenouda, Fabio Cury, Melissa Henry

**Affiliations:** ^1^ McGill University Montreal Quebec Canada; ^2^ Lady Davis Institute for Medical Research Montreal Quebec Canada; ^3^ Department of Psychology Tropical Futures Institute, James Cook University Singapore Singapore; ^4^ Department of Psychiatry and Dalla School of Public Health University of Toronto Toronto Ontario Canada; ^5^ McGill University Health Centre Montreal Quebec Canada; ^6^ Jewish General Hospital Montreal Quebec Canada

**Keywords:** cluster analysis, head and neck neoplasms, otolaryngology, quality of life, risk factors

## Abstract

**Background:**

Quality of life (QoL) in head and neck cancer (HNC) is influenced by complex biopsychosocial factors, yet few longitudinal studies have examined these relationships immediately post‐treatment.

**Methods:**

In this prospective study, 232 patients newly diagnosed with primary HNC completed psychometric assessments, clinical interviews, and medical chart reviews at baseline, 3, 6, and 12 months. QoL was measured using the Functional Assessment of Cancer Therapy–HNC module. A two‐step cluster analysis identified distinct biopsychosocial subgroups, and mixed ANOVA tested QoL trajectories across time.

**Results:**

Three clusters emerged: (1) early‐stage disease with low treatment intensity; (2) high psychological burden and (3) advanced disease with high treatment intensity. These were associated with highest QoL, persistently lowest QoL (*p* < 0.001), and intermediate QoL that improved over time respectively.

**Conclusions:**

Psychological distress exerted a stronger influence on QoL than medical burden. Early identification and intervention for psychosocial risk factors may optimize recovery in HNC patients.

## 1 Introduction

Head and neck cancer (HNC) and its treatments are known to impact quality of life (QoL) and functioning, significantly impacting bodily processes and structures, activities of daily living, and participation in life roles [1]. HNC can affect functional aspects such as food intake, breathing, speech, pain, mood, and shoulder‐neck mobility, with long‐term changes continuing to negatively impact QoL after cancer remission [2]. Moreover, the link between negative psychological factors, such as the presence of clinical depression, and decreased survival in HNC patients has been well established in the literature [3].

Few studies have longitudinally investigated a comprehensive model of biopsychosocial risk factors leading to QoL compromise immediately post‐treatment in patients with HNC. QoL predictors identified in previous longitudinal studies include medical comorbidities [4, 5], marital status [4], age [5], smoking status [4], alcohol [4], depression [6], physical functioning [6], tumor stage [4, 6, 7], lower socio‐economic status [5], unemployment [5], and feeding tube dependency [5]. On the other hand, physically active patients, and patients with a stable weight were less likely to experience dysphagia and eating restriction [8]. Large contemporary longitudinal cohorts have further demonstrated substantial heterogeneity in quality‐of‐life trajectories among patients with head and neck cancer. Studies from the Head and Neck 5000 cohort in the United Kingdom have shown that symptom burden, particularly cancer‐related fatigue, peaks in the early post‐treatment period and are strongly influenced by baseline depression, smoking status, disease stage, and multimodal treatment [9]. Similarly, the NET‐QUBIC cohort in the Netherlands has highlighted the persistent role of psychological factors, coping styles, and social resources in shaping long‐term supportive care needs, which are closely linked to overall quality of life, alongside clinical disease characteristics [10]. While these studies have identified important predictors of quality of life using predominantly variable‐centered approaches, less is known about how constellations of interacting biopsychosocial factors define distinct patient profiles with differential quality‐of‐life trajectories in the early survivorship period. The complex management of HNC with intent to improve quality of life may benefit from the further characterization of biopsychosocial risk profiles. Such profiles would identify patient groups who may be especially vulnerable to poor quality of life due to their combination of predisposing medical and psychosocial factors and may lead to the development of personalized interventions to mitigate risk and promote illness adaptation.

To guide this work, we used a conceptual model based on the Wilson‐Cleary model of QoL and the diathesis‐stress model. The Wilson‐Cleary model integrates psychological and biological domains, positing that QoL is determined by a complex array of biopsychosocial factors [11]. The diathesis‐stress model suggests that health is a result of an interaction between pre‐existing [12] vulnerabilities, or diathesis, and environmental stressors [13]. Our conceptual model included biomedical variables, present and past mental health, personality traits, sleep quality, and social support.

This study aimed to delineate biopsychosocial risk profiles associated with compromised QoL during the first year following a first occurrence of HNC. We used an unsupervised, machine learning, clustering algorithm to detect the presence of distinct patient profiles. We then examined these clusters concerning their trajectories of QoL over time and their relationship to medical and psychological factors. We hypothesized that psychological factors would be strongly associated with longitudinal QoL outcomes and would identify a subgroup of patients with persistently compromised QoL across the first year following diagnosis, independent of medical disease burden, based on our recent work within this population [14, 15, 16].

## 2 Materials and Methods

### 2.1 Design

This study employed a multicenter prospective longitudinal design. Structured Clinical Interviews for DSM‐IV Diagnoses (SCID‐I) [17] assessed major depressive disorder, anxiety disorders, and alcohol use disorder at baseline, 3‐month follow‐up, and lifetime pre‐cancer. Self‐administered psychometric measures were completed pre‐treatment (< 2 weeks of cancer diagnosis), and at 3, 6, and 12 months. This study received ethical approval from the McGill University Faculty of Medicine Institutional Review Board and recruiting hospitals (#A05‐B24‐10B). All participants provided informed consent.

### 2.2 Study Participants

Patients were eligible if they were adults (≥ 18 years), diagnosed with a primary HNC (TNM Classification System) [18] within 2 weeks of referral to study, and alert and capable of providing free and informed consent. They were ineligible if they presented an upon enrollment Karnofsky Performance Scale [19] score of less than 60 or a prognosis of less than 6 months according to medical judgment.

### 2.3 Procedure

Patients were recruited from the Department of Otolaryngology—Head and Neck Surgery at two teaching hospitals affiliated with McGill University: the Jewish General Hospital and McGill University Health Centre in Montreal, Canada. A consecutive record was maintained to track enrollment and participation over time. Following written informed consent, patients completed baseline questionnaires and were scheduled for an individual SCID‐I interview. Follow‐up questionnaires were sent via email with three weekly reminders, along with a self‐addressed, pre‐stamped envelope.

### 2.4 Outcome Measure

The Functional Assessment of Cancer Therapy—General and Head and Neck Module (FACT‐HN) [20] evaluated participants' QoL over the past week (internal validity: 0.89; test–retest reliability: 0.92).

### 2.5 Predictor Measures

The initial intake questionnaire captured basic sociodemographic information at baseline (i.e., age, sex, education, marital status, living alone), cigarette smoking and prior suicidal ideation/attempts. Medical variables were assessed through medical chart reviews at every timepoint throughout the study (i.e., cancer stage, cancer site, anticancer treatment including free tissue transfer flap, radiation dose and chemotherapy agent, HPV‐status, ECOG Performance Status [19], PEG tube, disease recurrence or progression, time since last treatment).

Abuse in childhood (i.e., before age 12; physical, psychological, and sexual abuse, and neglect) was collected with questions from the Canadian Incidence Study of Reported Child Abuse and Neglect [21].

The SCID‐I assessed major depressive disorder, anxiety disorders, and alcohol use disorder at baseline, as well as lifetime pre‐cancer. The inter‐rater reliability for symptoms is 0.75, while the inter‐rater reliability for diagnoses, when training is provided, is 0.90 [17].

The Eastern Cooperative Oncology Group (ECOG) Performance Status [22] evaluated functional status. The Hospital Anxiety and Depression Scale (HADS) [23] measured psychological distress (internal consistency 0.78–0.93; test–retest reliability > 0.80). The Functional Assessment of Chronic Illness Therapy, Spiritual Wellbeing Scale (FACIT‐Sp) [24] assessed spiritual‐existential wellbeing (internal consistency > 0.74; test–retest reliability 0.91). The Brief COPE Scale [25] denial subscale was used to evaluate illness‐specific coping based on denial (internal consistency 0.84; test–retest reliability 0.72). The Eysenck Personality Questionnaire—Neuroticism Subscale (EPQ‐N) [26] measured neuroticism (internal consistency 0.83; test–retest reliability 0.92). The Social Support Questionnaire (SSQ) [27] assessed satisfaction and number of social supports (internal consistency 0.57; test–retest reliability 0.90).

The Social Readjustment Rating Scale (SRRS) [28] assessed the impact of major life events in the past year on participant. The Rapid Alcohol Problems Screen—Quantity Frequency (RAPS4‐QF) [29] assessed risk for alcohol‐related problems. The Body Image Scale (BIS) [30] measured body image symptoms (internal consistency 0.86–0.96; test–retest reliability > 70). The Pittsburgh Sleep Quality Index (PSQI) [31] measured sleep quality over the past month (internal consistency 0.79; test–retest reliability 0.91). The Charlson Comorbidity Index (CCI) [32] assessed the burden of comorbid conditions (internal consistency 0.63; test–retest reliability 0.91).

### 2.6 Statistical Analysis

Data were analyzed using SPSS Statistics (version 29.0). Data distributions respected parametric statistical assumptions. Expectation maximization was used to impute missing data on predictive measures; quality‐of‐life outcome data were not imputed using this single‐imputation approach. Longitudinal analyses of quality of life were conducted using linear mixed‐effects models with maximum likelihood estimation, which incorporate all available observations and do not require complete data at all timepoints. This approach allows participants with partially observed quality‐of‐life data to contribute information under a missing‐at‐random assumption [33]. Selection of variables for clustering was based on a backward elimination approach, using univariate tests (*T*‐tests, ANOVA, or Spearman's rho) with *p* < 0.01 as the significance criterion, with QoL at 3 month as the outcome variable. Variables demonstrating significance below cut‐off were considered for clustering. We used a two‐step cluster analysis to accommodate the mixture of categorical and continuous variables. This technique is versatile and widely used to identify subgroups of individuals based on distance measures and model‐based criteria. The two steps involve an initial pre‐clustering in many small subgroups, followed by merging these subclusters into a solution with an optimal number of clusters. Clinical variables with multiple categories (e.g., treatment modality, ECOG performance status, and cancer stage) were included in the unsupervised clustering algorithm as categorical indicators reflecting overall disease burden and treatment intensity. These variables were not entered as dummy‐coded predictors in regression or longitudinal models, but were used to characterize cluster composition descriptively rather than to support inferential comparisons at the level of individual categories.

After cluster identification, a mixed effects ANOVA was conducted testing for cluster differences over time in quality of life. Quality‐of‐life trajectories were examined as a function of cluster membership, not individual clinical subgroups. With a significant Cluster × Time effect, we then tested pairwise comparisons between clusters at each time point to explore the pattern of QoL differences across baseline, 3‐, 6‐, and 12‐month follow‐ups.

## 3 Results

### 3.1 Study Participants

Two‐hundred and thirty‐two out of 313 eligible patients participated (74% participation); and completed the FACT‐G and HN Module. Reasons for declining participation included insufficient time (33%), competing priorities (24%), and emotional‐ or time‐related concerns (39%). Attrition with time was not significantly impacted by sex, cancer stage, or baseline psychological distress on the HADS. Our study population represented 69.9% male, with a mean age of 63 years (range 30–101). Sixty percent were married, 30.5% living alone, and 35.1% obtained a university education. They mostly were diagnosed with advanced stage cancer (67.4%), with most predominant cancer sites being oropharynx (34.3%), oral (19.2%), larynx (15.5%), and hypopharynx (9.2%). Medical co‐morbidities were assessed and accounted for as confounders in our analyses. Other characteristics including medical treatments received can be found in Table 1.

**TABLE 1:** Sociodemographic, medical, and clinical characteristics of patients newly diagnosed with a first occurrence of head and neck cancer and their association with quality of life at 3 months post‐diagnosis.

Variables	*N* (%)	Mean (SD)	Association with quality of life at 3 months	Direction of association
Age		63.28 (11.40)	*r* = 0.14	No clear direction
			*p* = 0.10	
Marital status			*F* = 3.05	No clear direction
			*p* = 0.03*	
Married or common‐law	142 (61.2%)			
Divorced or separated	56 (24.1%)			
Single (never married)	22 (9.5%)			
Widowed	12 (5.2%)			
Sex			*t* = −0.09	No clear direction
			*p* = 0.93	
Female	69 (39.7%)			
Male	163 (70.3%)			
Living arrangement			*t* = −1.68	No clear direction
			*p* = 0.09	
Living alone	69 (29.7%)			
Living with someone	1663 (70.3%)			
Charlson comorbidity index at baseline		3.89 (1.15)	*r* = 0.12 *p* = 0.15	No clear direction
HPV status			*t* = 1.49	No clear direction
			*p* = 0.139	
Positive	90 (39.3%)			
Negative	142 (60.7%)			
Cancer site			*F* = 2.14	No clear direction
			*p* = 0.036*	
Oral	44 (19.0%)			
Nasopharynx	11 (4.7%)			
Oropharynx	81 (34.9%)			
Hypopharynx	22 (9.5%)			
Larynx	36 (15.5%)			
Paranasal sinuses and nasal cavity	5 (2.2%)			
Salivary glands	6 (2.6%)			
Skin	15 (6.5%)			
Thyroid	1 (0.4%)			
Unknown primary	11 (4.7%)			
Cancer stage			*F* = 6.56	↓ Stage III = IV > Stage I (Stage II not significantly different)
			*p* < 0.001***	
I	41 (17.7%)			
II	36 (15.5%)			
III	36 (15.5%)			
IV	119 (51.3%)			
Treatment type at 3 months post diagnosis			*F* = 4.81	↓ *R* + C = S + *R* + C > S (others not significantly different)
			*p* < 0.001***	
Surgery	3 (1.3%)			
Radiation	36 (15.5%)			
Chemotherapy	22 (9.5%)			
Surgery + radiation	20 (8.6%)			
Surgery + chemotherapy	23 (9.6%)			
Radiotherapy + chemotherapy	99 (42.7%)			
Surgery + radiation + chemotherapy	29 (12.5%)			
Education			*F* = 0.73	No clear direction
			*p* = 0.57	
Elementary	19 (8.2%)			
High School	72 (31.0%)			
CEGEP/Vocational	60 (25.9%)			
Undergraduate degree	40 (17.2%)			
Graduate degree	41 (17.7%)			
Feeding tube at 3 months post diagnosis			*F* = 14.26	↓ NG = PEG> None
			*p* < 0.001***	
None	81 (34.9%)			
Nasogastric (NG) tube	40 (17.2%)			
Percutaneous endoscopic gastrostomy (PEG) tube	111 (47.8)%			
Parental bonding			*F* = 1.21	No clear direction
			*p* = 0.30	
Optimal parenting	65 (28.0%)			
Affectionate constraint	161 (69.4%)			
Affectionless control	5 (2.2%)			
Neglectful	1 (0.4%)			
Free flap			*t* = 0.04	No clear direction
			*p* = 0.97	
Yes	28 (12.1%)			
No	204 (87.9%)			
Recurrent cancer at baseline			*t* = 0.76	No clear direction
			*p* = 0.45	
No recurrence or progression	229 (98.7%)			
Recurrence or progression	3 (1.3%)			
Cigarette in the last 30 days			*t* = 2.172	No clear direction
			*p* = 0.031*	
Yes	41 (17.7%)			
No	191 (82.3%)			
History of suicidal ideation			*t* = 1.64	No clear direction
			*p* = 0.1	
Yes	23 (0.9%)			
No	209 (90.1%)			
History of major depressive disorder—SCID			*t* = 2.37	↓ Past MDD>No past MDD
			*p* = 0.019*	
Yes	47 (20.3%)			
No	185 (79.7%)			
Mood, anxiety or substance use disorder at baseline—SCID			*t* = 1.91	No clear direction
			*p* = 0.058	
Yes	69 (29.7%)			
No	163 (70.3%)			
Presence of major Depressive disorder at baseline—SCID			*t* = 2.67	↓ Current MDD>No current MDD
			*p* = 0.009**	
Yes	14 (6.0%)			
No	218 (94%)			
Type of chemotherapy			*t* = 3.99	↓ Strong Chemotherapy>Weak
			*p* < 0.001***	
Strong: Carboplatin, taxol, or, cisplatin	106 (45.7%)			
Weak: docetaxel, cetuximab, panitumumabe, erbitux, mannitol	126 (54.3%)			
ECOG			*F* = 5.85	Overall association; direction not resolvable
			*p* < 0.001***	
Fully active	149 (64.2%)			
Restricted in physically strenuous activity	62 (26.7%)			
Unable to carry out any work activity	16 (6.9%)			
Capable of only limited self‐care	4 (1.7%)			
Cannot carry on any self‐care	1 (0.4%)			
Total radiotherapy dose at 3 months post diagnosis		1414.05 (874.52)	*r* = −0.43	↓ Higher radiation dose
			*p* < 0.001***	
Pittsburgh sleep questionnaire		7.86 (3.04)	*r* = −0.22	↓ Poorer sleep quality
			*p* = 0.01**	
Hospital anxiety and depression scale at baseline		9.59 (7.56)	*r* = −0.49	↓ Greater psychological distress
			*p* < 0.001	
Eysenck personnality inventory–neuroticism subscale		7.45 (6.07)	*r* = −0.37	↓ Higher neuroticism
			*p* < 0.001***	
COPE denial subscale		3.07 (1.58)	*r* = −0.311	↓ Greater use of denial coping
			*p* < 0.001***	
COPE substance use subscale		2.30 (0.91)	*r* = −0.23	↓ Greater substance use coping
			*p* = 0.0068**	
Social support questionnaire—satisfaction with supports subscale		33.15 (5.32)	*r* = 0.22	↑ Greater satisfaction with social support
			*p* = 0.009**	
Body image scale		4.26 (5.54)	*r* = −0.26	↓ Greater body image disturbance
			*p* = 0.002**	
FACIT—spiritual wellbeing		28.21 (7.22)	*r* = 0.29	↑ Greater spiritual wellbeing
			*p* < 0.001***	
Rapid alcohol problems screen		1.46 (1.34)	*r* = 0.03	No clear direction
			*p* = 0.74	

*Note:* ↓ indicates lower quality of life; “>” indicates worse quality of life at 3 months; “=” indicates no significant difference between groups. ↑ indidcates higher quality of life. No clear direction indicates that although the omnibus test was statistically significant, no post hoc pairwise comparisons were significant after Bonferroni correction. For ECOG performance status, post hoc pairwise comparisons were not performed due to sparse cell counts in higher ECOG categories (*n* < 2 in at least one group), precluding resolution of directionality (*n* = 232). *p < 0.05; **p < 0.01; ***p < 0.001.

Abbreviations: SCID, Structured Clinical Interview for DSM‐IV Diagnoses; COPE, Coping Orientation to Problems Experienced Inventory; FACIT, Functional Assessment of Chronic Illness Therapy; ECOG, Eastern Cooperative Oncology Group Performance Status.

### 3.2 Cluster Definition

Table 1 provides an overview of sample characteristics and their associations with 3‐month QoL. Variables that met a threshold of *p* < 0.01 were include in the subsequent cluster analysis [32]: cancer stage, functional status, treatment modality, use of chemotherapy, radiation dose, feeding tube dependency, time elapsed since treatments, major depressive disorder at diagnosis, anxiety symptoms (HADS anxiety subscale), depressive symptoms (HADS depression subscale), psychological distress (HADS Total), neuroticism, sleep quality, denial, substance use, body image, and spirituality. The cluster analysis produced a three‐cluster solution (see Table 2 for cluster characterization).

**TABLE 2:** Key variables identified for cluster analysis and their respective average values across all patients of the three clusters.

Variable	Cluster 1	Cluster 2	Cluster 3
	(Low intensity)	(High psychological risk)	(High intensity)
	Mean (SD)	Mean (SD)	Mean (SD)
	*N* (%)	*N* (%)	*N* (%)
	Characterized by low intensity treatment and reduced psychological risk factors	Characterized by elevated psychological risk factors	Characterized by advanced disease and elevated treatment intensity
Total radiation dose at 3 months (Gy)	604.15 (704.07)	1465.89 (758.08)	1985.07 (874.52)
Time since last treatment (days)	57.78 (26.78)	47.95 (24.92)	24.30 (20.06)
HADS total score	6.33 (4.84)	17.92 (7.52)	6.84 (4.94)
HADS anxiety subscale	4.50 (3.47)	10.19 (4.46)	4.42 (3.31)
HADS depression subscale	1.83 (1.96)	7.72 (3.98)	2.42 (2.65)
EPQR neuroticism subscale	5.40 (4.65)	13.18 (6.19)	5.41 (4.46)
COPE denial subscale	2.66 (1.25)	3.98 (1.86)	2.81 (1.41)
COPE substance use subscale	2.20 (0.86)	2.45 (0.78)	2.29 (1.00)
Social support questionnaire—satisfaction with supports subscale	34.81 (2.87)	29.86 (6.75)	33.96 (4.84)
Body image scale	2.68 (3.37)	7.96 (7.13)	3.14 (4.23)
FACIT‐spiritual wellbeing–total score	30.50 (6.23)	24.49 (7.11)	28.83 (7.13)
Treatment at 3 months post diagnosis			
Surgery	3 (100%)	0	0
Radio	31 (86.1%)	5 (13.9%)	0
Chemo	18 (81.8%)	4 (18.2%)	0
S + *R*	0	18 (90%)	2 (10%)
S + C	20 (87%)	3 (13%)	0
C + *R*	0	27 (27.3%)	72 (72.7%)
S + C + *R*	1 (3.4%)	4 (13.8%)	24 (82.8%)
ECOG			
0	63 (42.3%)	12 (8.1%)	74 (49.7%)
1	6 (9.7%)	37 (59.7%)	19 (30.6%)
2	3 (18.8%)	8 (50%)	5 (31.3%)
3	1 (25%)	3 (75%)	0
4	0	1	0
Feeding tube			
No	63 (77.8%)	9 (11.1%)	9 (11.1%)
NG	8 (20%)	26 (65%)	6 (15%)
PEG	2 (1.8%)	26 (23.4%)	83 (74.8%)
SCID—Major depressive disorder at diagnosis			
No	73 (33.5%)	49 (22.5%)	96 (44%)
Yes	0 (0%)	12 (85.7%)	2 (14.3%)
Chemotherapy type			
Strong: Carboplatin, Taxol or Cisplatin	0 (0%)	22 (20.8%)	84 (79.2%)
Weak: Docetaxel, Cetuximab, Panitumumabe, Erbitux, Mannitol	73 (57.9%)	39 (31%)	14 (11.1%)
Cancer stage			
1	39 (95.1%)	2 (4.9%)	0 (0%)
2	14 (38.9%)	21 (58.3%)	1 (2.8%)
3	7 (19.4%)	12 (33.3%)	17 (47.2%)
4	13 (10.9%)	26 (21.8%)	80 (67.2%)

*Note: n* = 232 (Cluster 1 *n* = 73, Cluster 2 *n* = 61, Cluster 3 *n* = 98).

Abbreviations: COPE, Coping Orientation to Problems Experienced Inventory; ECOG, Eastern Cooperative Oncology Group Performance Status; EPQ‐R, Eysenck Personality Questionnaire‐Revised; FACIT, Functional Assessment of Chronic Illness Therapy; HADS, Hospital Anxiety and Depression Scale; SCID, Structured Clinical Interview for DSM‐IV Diagnoses.

#### 3.2.1 Cluster 1—Early Stage and Low Intensity Treatment

Cluster 1 (*N* = 73) included 31.5% of patients and stood out as marked by early‐stage disease, low‐intensity treatment, and fewer psychological disturbances. These individuals represented 95% of Stage 1 cancer. None of the patients in this cluster needed chemotherapy, 86% did not require a feeding tube, and their mean radiation dose was significantly lower than clusters 2 and 3 (*p* < 0.05) contributing to their low‐intensity treatment profile. Additionally, major depression upon diagnosis was absent within this cluster.

#### 3.2.2 Cluster 2—High Psychological Burden

Cluster 2 (*N* = 61) comprised 26.3% of the cohort, representing patients with moderate‐intensity disease and treatment indices, coupled with elevated baseline psychological diagnoses and higher psychological distress. Sixty‐three percent presented with early‐stage cancer, 36% required chemotherapy, 43% a nasal‐gastric and 43% a percutaneous endoscopic gastrostomy (PEG) feeding tube, contributing to the increased treatment intensity as compared to cluster 1. Notably, 85% of those exhibiting major depression at diagnosis were grouped within this cluster, reflecting the effect of psychological distress in cluster identity.

#### 3.2.3 Cluster 3—Advanced Disease and High Intensity Treatment

Cluster 3 (*N* = 98) included 42.2% of study participants and was characterized by advanced disease and high‐intensity treatment. A significant proportion (62.5%) had advanced‐stage cancer in this cluster and most required chemotherapy at some point in their treatment regimen (86%), underlining the disease severity and treatment intensity. This group exhibited the highest mean radiation dose and 85% required a gastric feeding tube. However, they shared similar psychological distress profiles as Cluster 1.

### 3.3 Visualization of Cluster Profiles and Predictor Importance

**FIGURE 1:**
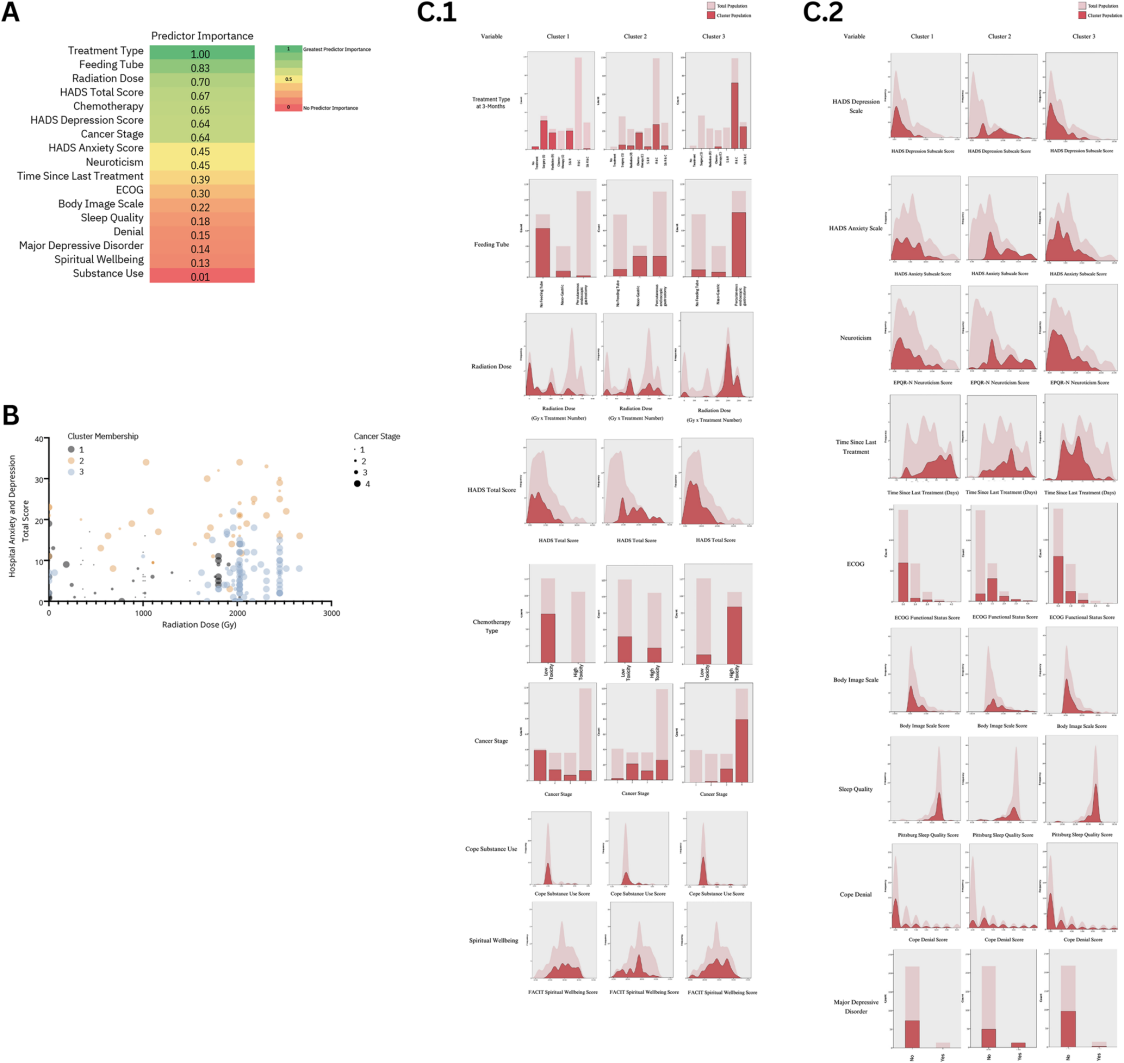
Key Variables and Their Relative Importance Per Cluster. (A) Variables included in the cluster analysis and how influential each variable is in determining the clusters. (B) Bubble plot depicting the three clusters in different colors and their relationship to psychological factors (y‐axis), treatment paradigms (x‐axis) and disease state (bubble size). (C.1 & C.2) For continuous variables histograms show the frequency distribution of the variable values within each cluster, with the cluster population show in dark red and the total population in pink. The x‐axis represents the range of variable values, while the y‐axis represents the frequency or count of cases. For categorical variables, bar charts display the proportion or count of each category within each cluster, with the cluster population show in dark red and the total population in pink. The x‐axis represents the categories, while the y‐axis represents the proportion or count of cases in each category. Chemotherapy (C) S & R & C No Treatment Surgery (S) Radiation (R) Chemotherapy (C) S & R R & C S& R & C; *n* = 232. [Color figure can be viewed at wileyonlinelibrary.com]

Figure 1A shows the predictor importance of each variable in delineating cluster membership. A distinct set of variables emerged as key drivers in shaping the clusters, underlining their roles in characterizing patient subgroups. The variables exerting the greatest influence on cluster formation in order of importance were treatment type, feeding tube requirement, radiation dose, psychological distress (HADS Total), chemotherapy utilization, depressive symptoms (HADS depression subscale), and cancer stage.

The bubble map, Figure 1B, was used to visually represent the three clusters across the identified key biopsychosocial dimensions in Figure 1A and the key characteristics of each cluster. This map illustrates the interplay of continuous and categorical variable for cluster membership.

Cluster 1 (Gray) is positioned in the lower range of radiation dose, psychological distress scores, and cancer stage. Cluster 2 (Orange) occupies a middle range of radiation dose and cancer stage and has the highest psychological distress scores. Cluster 3 (Blue) is characterized by lower psychological distress scores, advanced cancer stage, and high radiation dose.

Figure 1C.1,C.2 depicts the distribution of patients within each cluster for individual variables, providing a snapshot of specific characteristic prevalence within each cluster and further emphasizing the distinct profiles.

### 3.4 Quality of Life Outcomes Over Time

**FIGURE 2:**
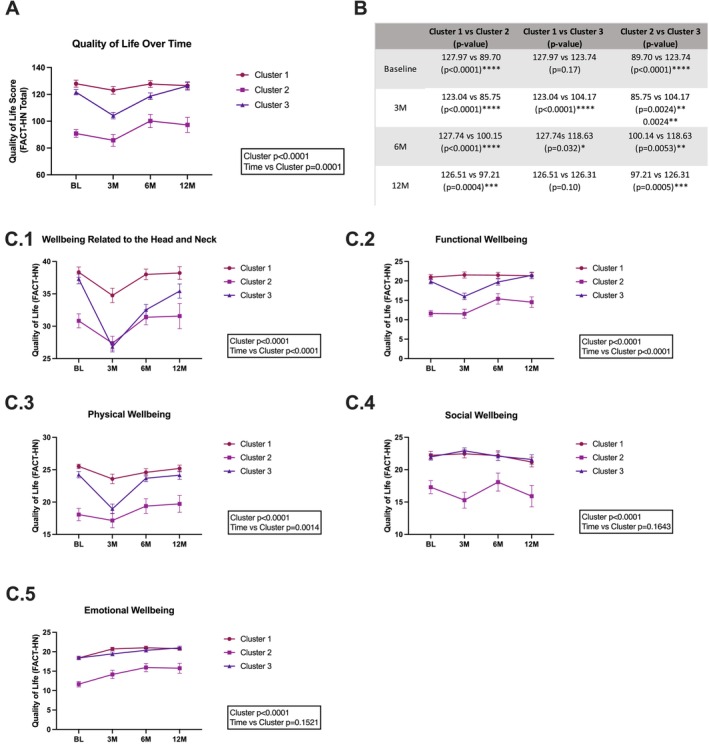
Quality of Life Per Cluster Over Time. (A) Total quality of life score at baseline, 3 months, 6 months and 1 year after a newly diagnosed head and neck cancer for each cluster. (B) Mean total quality of life scores per cluster at each time point. Mixed effects ANOVA with Bonferroni adjusted comparisons. (C): Mean quality of life subscale scores over time in relation to cluster membership at baseline, 3 months, 6 months and 1 year after diagnosis for: Head and Neck Cancer Subscale (C1), Functional Wellbeing Subscale (C2), Physical Wellbeing Subscale (C3), Social Wellbeing Subscale (C4) and Emotional Wellbeing Subscale (C5); *n* = 232. [Color figure can be viewed at wileyonlinelibrary.com]

Quality‐of‐life data were available for 210 participants at baseline, 140 at 3 months, 136 at 6 months, and 108 at 12 months. A mixed effects ANOVA indicated a statistically significant Cluster × Time interaction (*F*(6, 339) = 4.672, *p* < 0.001). Figure 2A,B summarizes total QoL scores per cluster at each time point; with significant differences in QoL values between clusters at 3‐(*p* < 0.0001) and 6‐months (*p* < 0.01), with most marked differences at 3 months. Significant differences in total QoL outcomes immediately post‐treatment (i.e., 3 months post‐diagnosis) were observed between all 3 clusters (Figure 2A). Cluster 3, characterized by the highest treatment intensity, displayed the most pronounced reduction in total QoL scores from baseline to 3 months. Cluster 2, characterized by greater psychological risk factors, consistently exhibited the lowest QoL scores across all four time points, with its lowest QoL score observed at 3 months. While Clusters 1 and 3 returned to healthy levels with time, Cluster 2 did not. These findings indicate that psychological variables are associated with persistently poorer quality of life across the first year following diagnosis, whereas the magnitude of acute QoL decline and subsequent recovery appears more closely aligned with treatment‐related medical burden, particularly among patients receiving high‐intensity therapy.

When sub‐domains of QoL were investigated over time (Figure 2C1–C5), QoL scores in Cluster 2 are consistently lower in all areas and are not driven by a single factor, namely in head and neck cancer related factors (Figure 2C1), functional wellbeing (Figure 2C2), and domains of physical wellbeing (Figure 2C3), social wellbeing (Figure 2C4), and emotional wellbeing (Figure 2C5). For Cluster 3, the reduction in QoL at 3 months was driven by the physical and the head and neck sub‐domains.

## 4 Discussion

The present study aimed to delve into the dynamics of biopsychosocial factors and their influence on the QoL of individuals newly diagnosed with HNC. Examining data from a longitudinal design with a clustering approach, we aimed to improve our understanding of the interplay among medical, clinical, and psychosocial elements. These clusters showcased the diversity of individual contexts that people can experience, which arises from the interaction between psychosocial attributes and medical burden to affect healing and recovery. Our findings revealed three distinct patient clusters: Cluster 1 composed of Early‐Stage Disease and Low Intensity Treatment; Cluster 2 composed of High Psychological Burden; and Cluster 3 composed of Advanced Disease and High Intensity Treatment.

Cluster 1 members experienced the best quality of life throughout the cancer trajectory. This was to be expected as they had the lowest psychological and biomedical burden. However, in comparing cluster 2 and cluster 3, an interesting distinction in QoL outcomes was revealed. Indeed, while cluster 3 showed the most intense treatment and medical burden of the three clusters, it was cluster 2, characterized by substantial psychological difficulties, who consistently displayed the lowest QoL across all time points despite lesser medical burden. Cluster 3 showed the largest reduction in QoL (but still maintaining an absolute FACT‐HN score greater than cluster 2) at 3 months post‐diagnosis but was able to improve and reach a level of quality of life similar to Cluster 1 at 1 year. An important distinction emerging from these findings is between factors associated with acute change in QoL versus those associated with persistent impairment. Patients in Cluster 3, characterized by advanced disease and high‐intensity treatment, experienced the greatest decline in QoL immediately post‐treatment, consistent with the well‐established impact of treatment‐related toxicity. However, this group demonstrated substantial recovery across multiple QoL domains over time. In contrast, Cluster 2 patients, characterized by elevated psychological distress, showed consistently lower QoL scores across all time points, with limited recovery, despite a comparatively lower medical burden.

To further demonstrate this, we examined specific subcategories of QoL, including emotional, social, and physical wellbeing as well as factors related to head and neck function. Our analysis at the 3‐month timepoint revealed that physical wellbeing and head and neck symptom burden were similar between cluster 2 and 3, while social and emotional wellbeing were statistically different. In fact, social and emotional wellbeing showed very little change as compared to baseline in cluster 3. This suggests that cluster 3's improvement in QoL was due to their successful coping in the social and emotional domains. Pre‐existing psychosocial risk may exacerbate the effects of physical and functional deficits on the emotional and social aspects of quality of life.

Our findings are consistent with, and extend, results from large longitudinal cohort studies of head and neck cancer survivorship. Data from the Head and Neck 5000 study showed that quality‐of‐life–related symptoms such as cancer‐related fatigue worsen substantially in the early post‐treatment period and are independently associated with baseline depression, smoking, advanced stage, comorbidity, and multimodal treatment [9]. Similarly, long‐term follow‐up from the NET‐QUBIC cohort demonstrated that psychological factors (including anxiety, depression, fear of cancer recurrence, coping, and self‐efficacy) are among the most consistent determinants of persistent supportive care needs several years after treatment [10]. Recent longitudinal work also indicates that smoking status shapes quality‐of‐life recovery, with continued smokers showing limited improvement over time [34].

In contrast to these predominantly variable‐centered approaches, the present study used an unsupervised clustering framework to identify distinct biopsychosocial profiles integrating psychological vulnerability and medical burden. This approach revealed that patients characterized by high psychological risk experienced persistently poorer quality of life despite lower treatment intensity, whereas patients with high medical burden but lower psychological distress demonstrated substantial recovery over time. Together, these findings suggest that psychological vulnerability is not only a predictor of quality‐of‐life outcomes, as shown in prior cohorts, but may define a clinically meaningful subgroup at risk for sustained impairment during early survivorship. Notably, large cohort studies highlight strong contributions of medical burden (stage, multimodal treatment) and lifestyle factors (smoking) to longitudinal symptom and QoL outcomes; our findings align in demonstrating marked early decrements with higher treatment intensity, but suggest that psychological vulnerability may be particularly relevant for persistent impairment. Future work should validate these profiles in large longitudinal cohorts and test whether adding key symptom and lifestyle modifiers identified elsewhere (e.g., fatigue, fear of recurrence, smoking) refines profile‐based prediction of persistent quality‐of‐life impairment and identifies actionable targets for tailored prehabilitation interventions.

The existing literature has also shown that pre‐treatment depression and psychiatric history predicts post‐treatment depression and future suicidal ideation [14, 35]. Our study supports that such psychological vulnerabilities continue to contribute beyond disease and treatment characteristics to distress and quality of life outcomes in HNC. Psychological interventions may therefore have the potential to modify the QoL trajectory, potentially modifying disease recovery. QoL compromise has been widely acknowledged for its far‐reaching consequences on patient recovery with distress being associated with malnutrition [36], unemployment [37], and suicidal ideation [35]. Our findings resonate with these established connections, underlining the critical need for comprehensive psychosocial support as an integral component of HNC patient care and as a prophylactic treatment.

The development of targeted interventions could benefit from further clarification of the underlying mind–body pathways linking mental wellbeing, quality of life, and medical recovery. Recent work has shown that polygenic risk factors for depression predict physical symptom burden in patients with HNC (11). This finding aligns with hypotheses from psychoneuroimmunology concerning the connection between depression and pro‐inflammatory cytokines such as interleukin‐6 (IL‐6) and tumor necrosis factor‐alpha (TNF‐α) [38]. Chronic inflammation has been linked to structural and functional changes in brain regions involved in emotional processing and stress regulation [39]. Lastly, one can foresee future studies of gut‐microbiome and gut dysbiosis mechanisms contributing to mental health and quality of life in oncology, as dysregulation of inflammation through gut dysbiosis has been shown to play a role in depression, and human brain imaging studies confirm bidirectional communication between the gut and the brain, with gut microbiota composition linked to neural activity and brain structure [40]. These areas of scientific inquiry could lead to therapeutic innovation to support individuals newly diagnosed with HNC in combination with psychosocial and supportive care intervention.

### 4.1 Clinical Implications

Our study identifies three patient populations as it pertains to the impact of HNC on quality of life. It highlights how psychological distress impacts quality of life to a significant degree, such that patients report similar quality of life despite less intense illness markers and treatments. There may be a need to implement screening for distress protocols as well as integrated psycho‐oncology supports as part of pre‐habilitative programs to improve QoL outcomes in this patient population.

Such psychosocial assessments may be used to personalize interventions based on individual risks and needs. Tailoring support to address psychological distress early in the treatment process could potentially improve QoL outcomes and overall recovery. This approach highlights the need for a more holistic treatment strategy that considers not only the physical but also the psychological and social dimensions of patient care.

### 4.2 Study Limitations

While this study makes an important contribution to the literature, several limitations should be acknowledged. First, attrition was greater in Cluster 2, the subgroup characterized by elevated psychological burden. This differential loss to follow‐up may have resulted in an underrepresentation of individuals with persistent distress, potentially limiting generalizability and leading to conservative estimates of quality‐of‐life impairment in this group. Although attrition was greatest among participants in the high psychological risk cluster, longitudinal mixed‐effects models allowed inclusion of all available quality‐of‐life observations and reduced bias associated with incomplete follow‐up, under the assumption that data were missing at random.

Second, the use of self‐reported assessments, although common in QoL research, is susceptible to response bias and may have been affected by response shifts, where patients alter their definition of quality of life to adapt to the changes in their bodies.

### 4.3 Future Directions

Future research should continue to explore the biopsychological mechanisms underlying the relationship between psychological factors and QoL, including the potential roles of inflammation, gut‐brain communication, and other emerging areas of psychoneuroimmunology. Understanding these connections could lead to novel treatments aimed at enhancing mental health and QoL in HNC patients. This study used a novel method to detect and generate further hypotheses about moderating effects between biomedical and psychosocial domains, which would have been challenging to detect using traditional statistical methods. Future work may seek to further validate and improve upon this approach to detect clinically meaningful profiles.

In conclusion, this study highlights the critical need for a biopsychosocial approach in managing HNC, emphasizing that psychological support should be considered a cornerstone of comprehensive cancer care to optimize patient outcomes.

## Author Contributions

**Haley Deamond:** conceptualization, formal analysis, writing – original draft, writing – review and editing, visualization, and presentation of research. **Cyril Devault‐Tousignant:** writing – original draft, writing – review and editing, visualization, and presentation of research. **Melissa Henry:** conceptualization, methodology, investigation, writing – review and editing, supervision, project administration, and funding. **Jacob Lang:** conceptualization, methodology, and formal analysis. **Christopher Lo:** writing – review and, editing. **Jennifer Silver:** investigation, writing – review, and editing. **Nader Sadeghi:** investigation, writing – review, and editing. **Zeev Rosberger:** writing – review. **Saul Frenkiel:** investigation and writing – review. **Michael Hier:** investigation and writing – review. **Anthony Zeitouni:** investigation and writing – review. **Karen Kost:** investigation and writing – review. **Alex Mlynarek:** investigation and writing – review. **Keith Richardson:** investigation and writing – review. **Gabrielle Chartier:** investigation and writing – review. **Marco Mascarella:** investigation and writing – review. **Khalil Sultanem:** investigation and writing – review. **Georges Shenouda:** investigation and writing – review. **Fabio Cury:** investigation and writing – review.

## Funding

The author, Dr. Melissa Henry, disclosed receipt of the following financial support for the research, authorship, and/or publication of this article: This work was supported by the Fonds de Recherche du Québec‐Santé (FRQS) (grant number 24910).

## Conflicts of Interest

The authors declare no conflicts of interest.

## Data Availability

The data that support the findings of this study are available on request from the corresponding author. The data are not publicly available due to privacy or ethical restrictions.

## References

[1] M. Henry , J. G. Albert , S. Frenkiel , et al., “Body Image Concerns in Patients With Head and Neck Cancer: A Longitudinal Study,” Frontiers in Psychology 13 (2022): 816587. 35401366 10.3389/fpsyg.2022.816587 PMC8988682

[2] D. Dejaco , D. Riedl , S. Gasser , et al., “A Tool for Rapid Assessment of Functional Outcomes in Patients With Head and Neck Cancer,” Cancers (Basel) 13, no. 21 (2021): 5529. 34771691 10.3390/cancers13215529 PMC8582907

[3] A. A. Mäkitie , R. O. Alabi , L. Pulkki‐Råback , et al., “Psychological Factors Related to Treatment Outcomes in Head and Neck Cancer,” Advances in Therapy 41, no. 9 (2024): 3489–3519. 39110309 10.1007/s12325-024-02945-3 PMC11349815

[4] A. A. Osthus , A. K. Aarstad , J. Olofsson , and H. J. Aarstad , “Comorbidity Is an Independent Predictor of Health‐Related Quality of Life in a Longitudinal Cohort of Head and Neck Cancer Patients,” European Archives of Oto‐Rhino‐Laryngology 270, no. 5 (2013): 1721–1728. 23053388 10.1007/s00405-012-2207-0

[5] M. Wells , S. Swartzman , H. Lang , et al., “Predictors of Quality of Life in Head and Neck Cancer Survivors up to 5 Years After End of Treatment: A Cross‐Sectional Survey,” Supportive Care in Cancer 24, no. 6 (2016): 2463–2472. 26660345 10.1007/s00520-015-3045-6

[6] E. Hammerlid , E. Silander , L. Hornestam , and M. Sullivan , “Health‐Related Quality of Life Three Years After Diagnosis of Head and Neck Cancer—A Longitudinal Study,” Head & Neck 23, no. 2 (2001): 113–125. 11303628 10.1002/1097-0347(200102)23:2<113::aid-hed1006>3.0.co;2-w

[7] K. Bjordal , M. Ahlner‐Elmqvist , E. Hammerlid , et al., “A Prospective Study of Quality of Life in Head and Neck Cancer Patients,” Part II: Longitudinal Data. Laryngoscope 111, no. 8 (2001): 1440–1452. 11568582 10.1097/00005537-200108000-00022

[8] K. Färnqvist , K. Mälberg , A. Johar , A. Schandl , and P. Lagergren , “Trajectories of Patient‐Reported Outcomes After Oesophageal Cancer Surgery – A Population‐Based Study,” European Journal of Cancer 206 (2024): 114113. 10.1016/j.ejca.2024.114133 38797039

[9] L. Sharp , L. J. Watson , L. Lu , et al., “Cancer‐Related Fatigue in Head and Neck Cancer Survivors: Longitudinal Findings From the Head and Neck 5000 Prospective Clinical Cohort,” Cancers (Basel) 15, no. 19 (2023): 4864. 37835558 10.3390/cancers15194864 PMC10571913

[10] F. Jansen , D. Molenaar , Ö. Zarsat , et al., “Supportive Care Needs From Mid‐To Long‐Term Follow‐Up Among Head and Neck Cancer Survivors: A Longitudinal Cohort Study,” Psycho‐Oncology 34, no. 9 (2025): e70276. 40903428 10.1002/pon.70276 PMC12408419

[11] I. B. Wilson and P. D. Cleary , “Linking Clinical Variables With Health‐Related Quality of Life. A Conceptual Model of Patient Outcomes,” Journal of the American Medical Association 273, no. 1 (1995): 59–65. 7996652

[12] M. Benassi , S. Garofalo , F. Ambrosini , et al., “Using Two‐Step Cluster Analysis and Latent Class Cluster Analysis to Classify the Cognitive Heterogeneity of Cross‐Diagnostic Psychiatric Inpatients,” Frontiers in Psychology 11 (2020): 1085. 32587546 10.3389/fpsyg.2020.01085 PMC7299079

[13] S. M. Monroe and A. D. Simons , “Diathesis‐Stress Theories in the Context of Life Stress Research: Implications for the Depressive Disorders,” Psychological Bulletin 110, no. 3 (1991): 406–425. 1758917 10.1037/0033-2909.110.3.406

[14] M. Henry , F. Fuehrmann , M. Hier , et al., “Contextual and Historical Factors for Increased Levels of Anxiety and Depression in Patients With Head and Neck Cancer: A Prospective Longitudinal Study,” Head & Neck 41, no. 8 (2019): 2538–2548. 30887617 10.1002/hed.25725

[15] M. Henry , R. Harvey , L. M. Chen , et al., “Genetic Predisposition to Depression and Inflammation Impacts Symptom Burden and Survival in Patients With Head and Neck Cancer: A Longitudinal Study,” Journal of Affective Disorders 331 (2023): 149–157. 36948466 10.1016/j.jad.2023.03.007

[16] M. Henry , E. Sargi , S. Frenkiel , et al., “Longitudinal Study Indicating Antecedent Psychosocial Vulnerability as Predictor of Anxiety Disorders Post‐Treatment in People With Head and Neck Cancer,” Psycho‐Oncology 30, no. 11 (2021): 1910–1919. 34190381 10.1002/pon.5760

[17] M. B. S. R. First , M. Gibbon , and J. B. Williams , Structured Clinical Interview for DSM‐IV‐TR Axis I Disorders, Research Version, Non‐Patient Edition (SCID‐I/NP) (Biometrics Research, 2002).

[18] D. James , TNM Classification of Malignant Tumours (Wiley‐Blackwell, 2017) Brierley (Editor) MKGE, Christian Wittekind (Editor). 8th Edition. (978‐1‐119‐26357‐9).

[19] C. C. Schag , R. L. Heinrich , and P. A. Ganz , “Karnofsky Performance Status Revisited: Reliability, Validity, and Guidelines,” Journal of Clinical Oncology 2, no. 3 (1984): 187–193. 6699671 10.1200/JCO.1984.2.3.187

[20] M. A. List , L. L. D'Antonio , D. F. Cella , et al., “The Performance Status Scale for Head and Neck Cancer Patients and the Functional Assessment of Cancer Therapy‐Head and Neck Scale. A Study of Utility and Validity,” Cancer 77, no. 11 (1996): 2294–2301. 8635098 10.1002/(SICI)1097-0142(19960601)77:11<2294::AID-CNCR17>3.0.CO;2-S

[21] N. W. Trocmé , “Canadian Incidence Study of Reported Child Abuseand Neglect,” 2001 David. Maltreatment C, Epidemiology DoHSa.

[22] M. M. Oken , R. H. Creech , D. C. Tormey , et al., “Toxicity and Response Criteria of the Eastern Cooperative Oncology Group,” American Journal of Clinical Oncology 5, no. 6 (1982): 649–655. 7165009

[23] A. S. Zigmond and R. P. Snaith , “The Hospital Anxiety and Depression Scale,” Acta Psychiatrica Scandinavica 67, no. 6 (1983): 361–370. 6880820 10.1111/j.1600-0447.1983.tb09716.x

[24] A. H. Peterman , G. Fitchett , M. J. Brady , L. Hernandez , and D. Cella , “Measuring Spiritual Well‐Being in People With Cancer: The Functional Assessment of Chronic Illness Therapy—Spiritual Well‐Being Scale (FACIT‐Sp),” Annals of Behavioral Medicine 24, no. 1 (2002): 49–58. 12008794 10.1207/S15324796ABM2401_06

[25] C. S. Carver , “You Want to Measure Coping but Your Protocol's Too Long: Consider the Brief COPE,” International Journal of Behavioral Medicine 4, no. 1 (1997): 92–100. 16250744 10.1207/s15327558ijbm0401_6

[26] S. B. Eysenck and H. J. Eysenck , “An Improved Short Questionnaire for the Measurement of Extraversion and Neuroticism,” Life Sciences 1964, no. 3 (1962): 1103–1109. 10.1016/0024-3205(64)90125-0 14225366

[27] I. G. S. B. Sarason , E. N. Shearin , and G. R. Pierce , “A Brief Measure of Social Support: Practical and Theoretical Implications,” Journal of Social and Personal Relationships 4, no. 4 (1987): 497–510.

[28] T. H. Holmes and R. H. Rahe , “The Social Readjustment Rating Scale,” Journal of Psychosomatic Research 11, no. 2 (1967): 213–218. 6059863 10.1016/0022-3999(67)90010-4

[29] C. J. Cherpitel , “A Brief Screening Instrument for Problem Drinking in the Emergency Room: The RAPS4. Rapid Alcohol Problems Screen,” Journal of Studies on Alcohol 61, no. 3 (2000): 447–449. 10807217 10.15288/jsa.2000.61.447

[30] P. Hopwood , I. Fletcher , A. Lee , and G. S. Al , “A Body Image Scale for Use With Cancer Patients,” European Journal of Cancer 37, no. 2 (2001): 189–197. 11166145 10.1016/s0959-8049(00)00353-1

[31] D. J. Buysse , C. F. Reynolds, 3rd , T. H. Monk , S. R. Berman , and D. J. Kupfer , “The Pittsburgh Sleep Quality Index: A New Instrument for Psychiatric Practice and Research,” Psychiatry Research 28, no. 2 (1989): 193–213. 2748771 10.1016/0165-1781(89)90047-4

[32] M. E. Charlson , P. Pompei , K. L. Ales , and C. R. MacKenzie , “A New Method of Classifying Prognostic Comorbidity in Longitudinal Studies: Development and Validation,” Journal of Chronic Diseases 40, no. 5 (1987): 373–383. 3558716 10.1016/0021-9681(87)90171-8

[33] G. Verbeke and G. Molenberghs , Linear Mixed Models for Longitudinal Data (New York: Springer, 2000), http://site.ebrary.com/id/10002182.

[34] E. Mohebbi , A. L. Carr , G. Guthrie , et al., “Long‐Term Quality of Life in Head and Neck Cancer: The Role of Postdiagnosis Smoking Behavior,” Journal of Cancer Survivorship (2025).10.1007/s11764-025-01894-241115999

[35] M. Henry , Z. Rosberger , L. Bertrand , et al., “Prevalence and Risk Factors of Suicidal Ideation Among Patients With Head and Neck Cancer: Longitudinal Study,” Otolaryngology and Head and Neck Surgery 159, no. 5 (2018): 843–852. 10.1177/0194599818776873 29865939

[36] B. Britton , K. Clover , L. Bateman , et al., “Baseline Depression Predicts Malnutrition in Head and Neck Cancer Patients Undergoing Radiotherapy,” Supportive Care in Cancer 20, no. 2 (2012): 335–342. 21234608 10.1007/s00520-011-1087-y

[37] L. Broemer , M. Friedrich , G. Wichmann , et al., “Exploratory Study of Functional and Psychological Factors Associated With Employment Status in Patients With Head and Neck Cancer,” Head & Neck 43, no. 4 (2021): 1229–1241. 33615608 10.1002/hed.26595

[38] R. Dantzer , J. C. O'Connor , G. G. Freund , R. W. Johnson , and K. W. Kelley , “From Inflammation to Sickness and Depression: When the Immune System Subjugates the Brain,” Nature Reviews. Neuroscience 9, no. 1 (2008): 46–56. 18073775 10.1038/nrn2297 PMC2919277

[39] A. H. Miller and C. L. Raison , “The Role of Inflammation in Depression: From Evolutionary Imperative to Modern Treatment Target,” Nature Reviews. Immunology 16, no. 1 (2016): 22–34. 10.1038/nri.2015.5 PMC5542678 26711676

[40] Z. A. Barandouzi , A. R. Starkweather , W. A. Henderson , A. Gyamfi , and X. S. Cong , “Altered Composition of Gut Microbiota in Depression: A Systematic Review,” Frontiers in Psychiatry 11 (2020): 541. 32587537 10.3389/fpsyt.2020.00541 PMC7299157

